# Simultaneous emission of Gaussian-like and parabolic-like pulse waveforms in an erbium-doped dual-wavelength fiber laser

**DOI:** 10.1038/s41598-017-09889-5

**Published:** 2017-08-25

**Authors:** Xing Li, Shenggan Dai, Weiwen Zou, Jianping Chen, Qiuhua Nie, Shixun Dai

**Affiliations:** 1 Laboratory of Infrared Materials and Devices, The Research Institute of Advanced Technologies, Ningbo University, Ningbo, 315211 China; 2 Key Laboratory of Photoelectric Materials and Devices of Zhejiang Province, Ningbo, 315211 China; 3 State Key Laboratory of Advanced Optical Communication Systems and Networks, Department of Electronic Engineering, Shanghai Jiao Tong University, Shanghai, 200240 China

## Abstract

We report on the observation of different pulse formation dynamics in a nonlinear polarization evolution (NPE)-based broadband erbium-doped fiber laser when the net cavity group-velocity dispersion (GVD) is managed to be close to zero. The fiber laser can generate pulses with a single wavelength or dual wavelengths by adjusting the waveplates. When the laser operates in dual-wavelength emission, the output pulses corresponding to the two wavelengths exhibit Gaussian- and parabolic-like waveforms, respectively, indicating that the laser can simultaneously operate in stretched-pulse and self-similar regimes. The generation of dual-wavelength emission with different pulse waveforms can be attributed to an overdriven NPE switch acting on a chirped broadband pulse and different dispersion mechanisms. These findings provide a good foundation for comprehensively studying pulse formation dynamics in laser cavities.

## Introduction

In the past two decades, passively mode-locked fiber lasers play important roles in numerous applications due to their significant advantages of excellent stability, low cost, easy operation, and compact design^1–7^. The development of the fiber lasers has been considerably advanced by the discovery of new mode-locking mechanisms. Based on the net cavity group-velocity dispersion (GVD), four distinct operating regimes (including conventional soliton^8^, stretched-pulse^9^, self-similar^10^, and all-normal dispersion regimes)^11^ have been implemented in mode-locked fiber lasers. The physics of mode-locked fiber lasers displays a rich interplay of dispersion, nonlinear, gain and absorption effects; such fiber lasers thereby provide a convenient experimental platform for comprehensively studying the pulse formation dynamics in laser cavities.

It has been theoretically and experimentally confirmed that pulse formation dynamics in the laser cavity are complex when the net cavity GVD is located in the transition between different operating regimes^12–18^. For example, B. Oktem *et al*.^16^ reported a new mode-locking regime where solitons and similaritons are emitted at different positions of the same laser with a net GVD of 0.0136 ps^2^. A Tünnermann *et al*.^17^ indicated that dispersion-managed solitons and similaritons can be switched in a net positive-dispersion cavity by adjusting the gain parameter based on theoretical study and simulation. J. Peng *et al*.^18^ showed that dissipative solitons, similaritons, and dispersion-managed solitons can be observed in a NPE-based normal-dispersion fiber laser depending on pulse intensity, linear phase delay and fiber birefringence of the laser cavity.

So far, the study of pulse formation dynamics has been limited to a single mode-locking regime while simultaneous emission of dual-wavelength pulses with different laser operating regimes in a laser cavity has been elusive. In this paper, we report on the experimental observation of two different pulse formation dynamics in a NPE-based erbium-doped (Er-doped) fiber ring laser at near zero cavity dispersion. By adjusting the waveplates to alter the intracavity polarization state, it is possible to switch output pulses from single-wavelength emission to dual-wavelength emission. The two pulse waveforms corresponding to the dual-wavelength emission exhibit distinct spectral profiles in leading and trailing edges. The shorter-wavelength pulses at 1533.1 nm operate in self-similar regimes while the longer-wavelength pulses at 1615.4 nm operate in stretched-pulse regimes. The formation of the dual-wavelength emission with different pulse waveforms results from the combination of an overdriven NPE switch and different dispersion mechanisms in the laser cavity. This is the first demonstration of coexistence of two different laser operating regimes in a NPE-based ultrafast fiber laser.

## Results and Discussions

### Setup of mode-locked fiber laser

**Figure 1:**
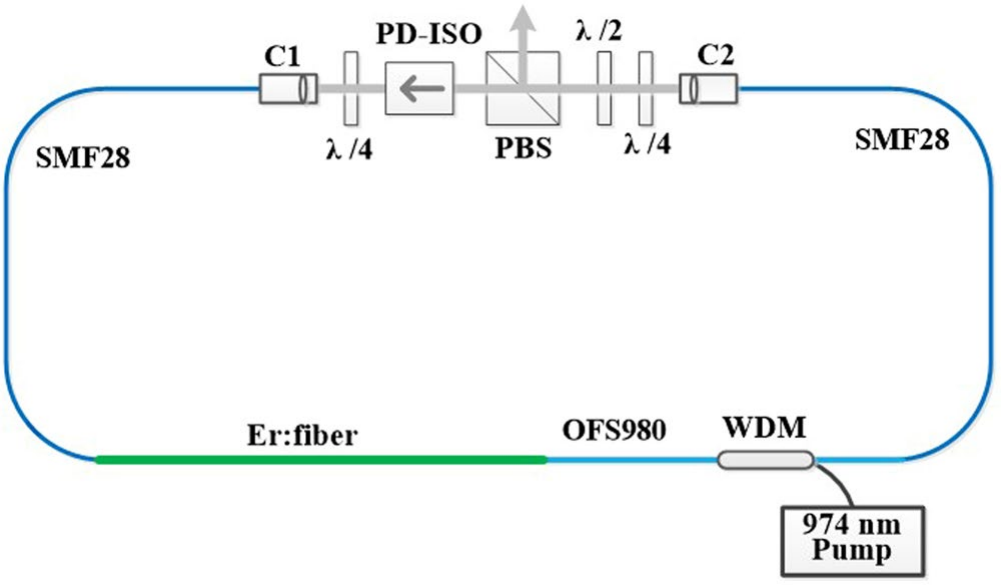
Configuration of the NPE-based dual-wavelength mode-locked fiber laser. PBS, polarization beam splitter; ISO, polarization-dependent isolator; λ/2, half waveplate; λ/4, quarter waveplate; WDM, wavelength-division multiplexer.

As shown in Fig. 1, the basic experimental setup for dual-wavelength emission investigated in this work is a typical NPE-based mode-locked ring fiber laser^7, 16^. The net cavity GVD can be controlled through appropriately selecting fibers with different lengths and dispersion values. Considering the GVD of PD-ISO (~0.003 ps^2^) and other free-space components (~−0.00042 ps^2^, including the three waveplates and the PBS), the net cavity GVD is optimally managed at +0.0048 ps^2^, which is close to zero at 1550 nm. The optimized lengths, GVD coefficients and nonlinear coefficients (N) of all fibers in the cavity are illustrated in Table 1.

**Table 1:** Parameters of all fibers in the NPE mode-locked cavity.

Length (mm)		Er:fiber	Nufern 980	Collimator1	Collimator2
		380	320	70	110
GVD (fs^2^/mm)	1550 nm	11.8	4.5	−20.6	−20.6
	1533 nm	12.9	5	−19.4	−19.4
	1615 nm	8.3	3.2	−24.9	−24.9
N (W^−1^/m)		0.0032	0.0021	0.0012	0.0012

The laser output is characterized by a commercial frequency-resolved optical gating (FROG, Grenouille & Frog15–40) and an optical spectrum analyzer (Yokogawa AQ6375, resolution 0.05 nm). The output pulse is detected by a 12.5-GHz photodetector (Newport, 818-BB-51F), and then analyzed by a 1-GHz real-time oscilloscope (Agilent infiniium 54833D) and an electrical spectrum analyzer (Agilent N9000B CXA).

### Mode-locking operation and characteristics

**Figure 2:**
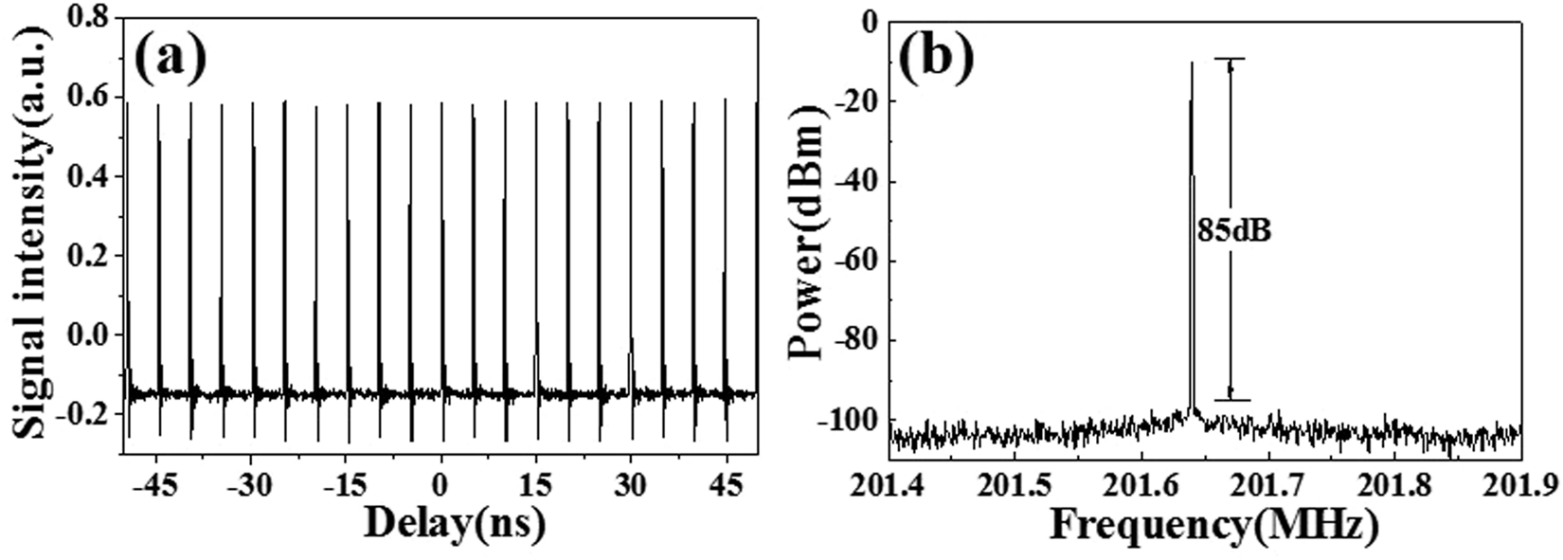
(**a**) Output pulse trains measured by an oscilloscope. (**b**) RF spectrum of fundamental mode beat (resolution bandwidth, 300 Hz).

The NPE-based mode-locking can be easily initialized by adjusting the waveplates when the pump power is above the threshold of 320 mW. Once set, the single pulse operation is self-starting and highly stable. When the pump power is increased to 600 mW, the maximum average output power of the mode-locked pulse is 75 mW, indicating a transfer efficiency of 12.5%. Figure 2(a) illustrates equally spaced pulses emitted from the laser with a fundamental repetition rate of larger than 200 MHz, suggesting no signs of dual-pulsing or Q-switched mode-locking operation of the laser. The slight fluctuation in pulse amplitude can be attributed to insufficient bandwidth and resolution of the oscilloscope. The RF spectrum of the fundamental mode beat is illustrated in Fig. 2(b). The signal-noise-ratio of the fundamental frequency is up to 85 dB at a resolution of 300 Hz, and no sidebands are observed within a 0.5 MHz frequency range.

**Figure 3:**
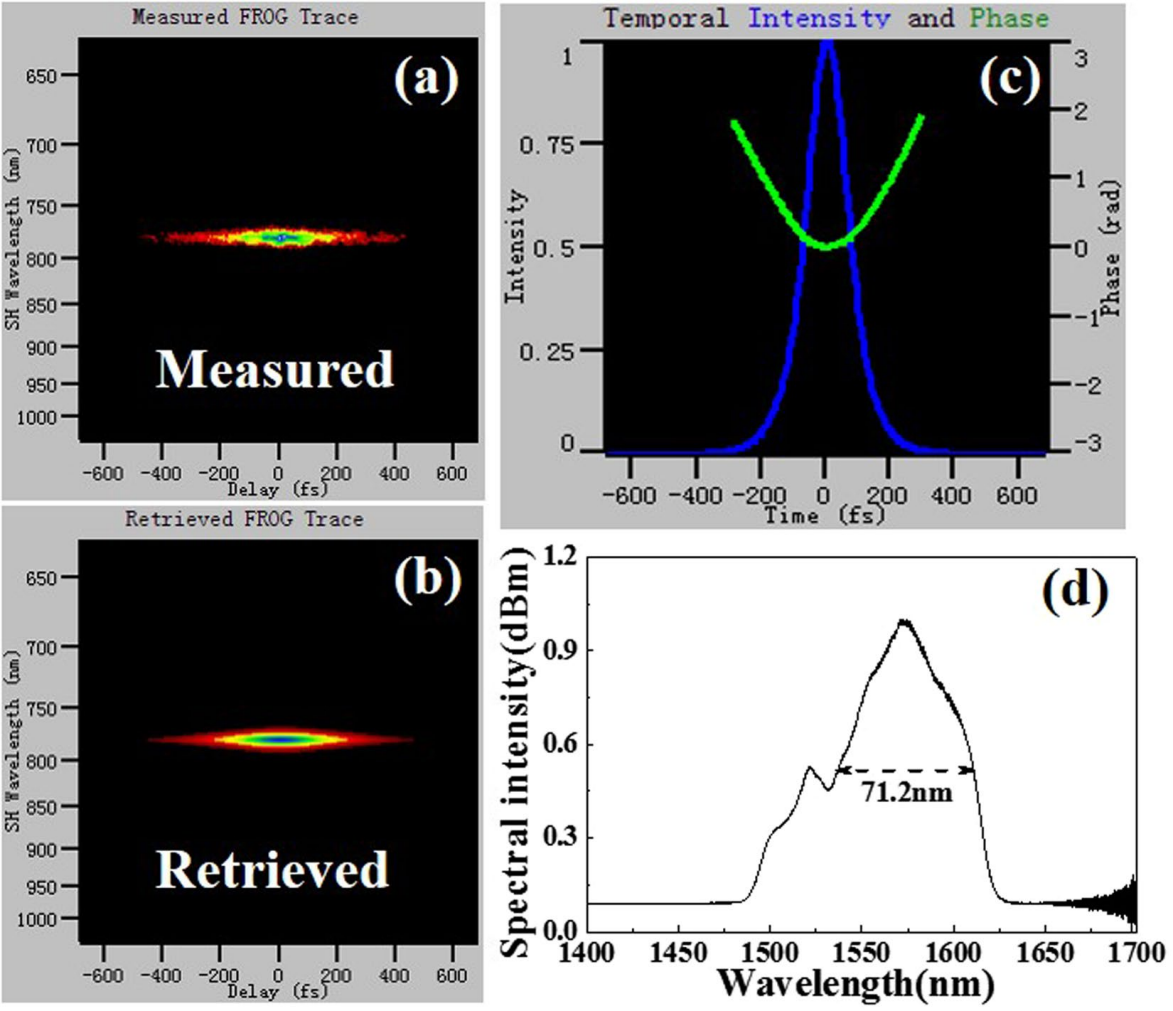
(**a**) Measured FROG trace, (**b**) retrieved FROG trace, (**c**) pulse and phase in time domain retrieved from FROG trace, and (**d**) spectrum when the laser is operated in single-wavelength emission.

The measured and retrieved FROG traces of the direct output pulses are illustrated in Fig. 3(a) and (b), respectively. Figure 3(c) presents the pulse intensity and phase in time domain retrieved from Fig. 3(a). It is noted that the phase quadratic factor represents a linear frequency chirp, which is a typical characteristics of self-similar solitons. That is to say, the pulse intensity exhibits a parabolic profile with a pulse duration of 153.6 fs. The corresponding spectrum in Fig. 3(d) shows a center wavelength of 1574.4 nm and a full width at half maximum (FWHM) of 71.2 nm (from 1538.3 nm to 1609.5 nm). The time-bandwidth product of the pulses is 1.367, which is much higher than the Fourier transform limit of 0.441, suggesting the presence of a large linear chirp. Besides, an obvious concavity at 1530 nm appears (Fig. 3(d)), which is possibly due to the fact that the ground state absorption in the gain fiber is not fully pumped by 980 nm light^19^.

**Figure 4:**
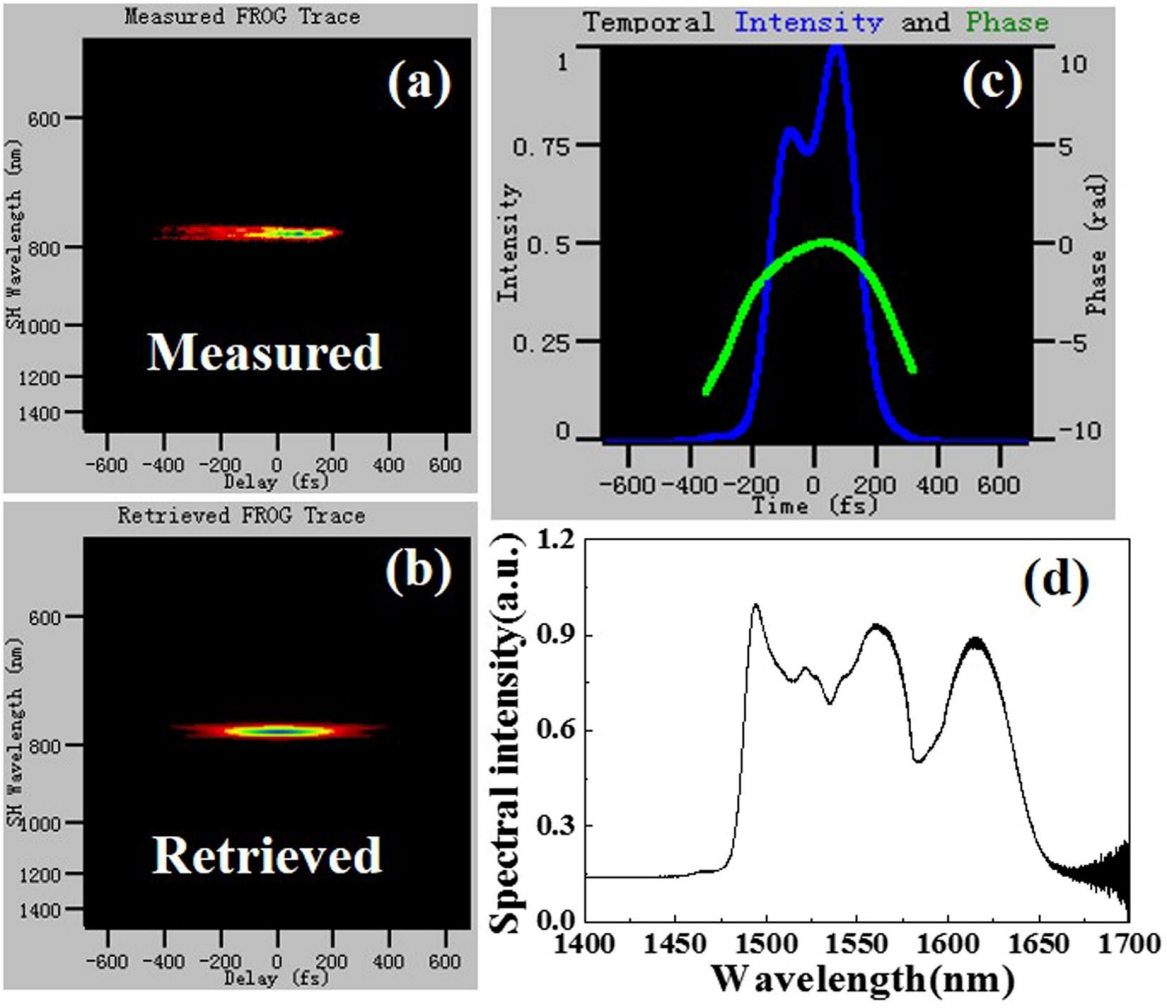
(**a**) Measured FROG trace, (**b**) retrieved FROG trace, (**c**) pulse and phase in time domain retrieved from FROG trace, and (**d**) spectrum when the laser is operated in dual-wavelength emission.

When the pump power is maintained at 600 mW and the waveplates in the cavity are finely adjusted, the single mode-locked pulse can be split into two partially overlapped pulses. Figure 4(a) and (b) are the measured and retrieved FROG traces, respectively. Figure 4(c) represents the pulse and phase in time domain which are retrieved from Fig. 4(a) and (b). The pulse waveform with a pulse duration of 191.6 fs demonstrates double peaks with partial overlap in intensity. In comparison, the phase of the primary pulse indicates a linear chirp whereas that of the secondary pulse suffers a little distortion. The output spectrum is very broad with a FWHM of about 145.4 nm (from 1488.1 nm to 1633.5 nm, see Fig. 4(d)). There are three peaks at the top of the spectrum, and the peak wavelengths are 1494.2 nm, 1560.6 nm and 1615.4 nm, respectively. The local minimum (~1530 nm) between the peak wavelengths of 1494.2 nm and 1560.6 nm is caused by the ground state absorption of the long gain fiber. In fact, there are only two spectra centered at 1533.1 nm and 1615.4 nm (the center wavelength of the FWHM spectral range), and the spectrum centered at 1533.1 nm is more energetic than that at 1615.4 nm. That is to say, fiber laser outputs a modulated spectrum with a ∼4 dB local minimum at 1582.5 nm and two temporally offset parts with different intensities. The FWHM of two spectra centered at 1533.1 nm and 1615.4 nm are 93.1 and 42 nm, respectively. There are many factors contributing to the broadband spectrum. On the one hand, fiber lasers based on NPE can generate a broad spectrum due to the large modulation depth and essentially instantaneous response. On the other hand, setting the total cavity dispersion to zero and reducing the effective cavity nonlinearity are verified as a better way to generate a broadband spectrum and thereby ultrashort pulses. It is noted that the leading edge of the spectrum is steeper than the trailing edge, indicating that the two spectra have different spectral characteristics. By using an additional spectral filter outside the cavity, it is manifested that the primary pulse corresponds to shorter wavelength spectrum of 1533.1 nm, while the secondary pulse corresponds to longer wavelength spectrum of 1615.4 nm. Therefore, the two pulses have different pulse characteristics in both frequency and time domains. In addition, the wavelength spacing between two spectra is 82.3 nm, and the pulse separation in time domain is 102 fs, indicating that the product of the spacing of the spectrum modulation (converted to the unit of frequency) and the pulse separation in time domain is approximately 1. The average output power of the dual-wavelength emission is 48 mW, which is lower than that of the single-wavelength emission. The dual-wavelength emission is stable provided that there are no large changes in polarization which alters the cavity birefringence. Here it works well under 24-hour continuous monitoring. The dependence of the dual-wavelength emission on incavity waveplates orientation is also investigated to further optimize the laser output characteristics. Although some changes in spectrum width, intensity and spacing are observed in the spectral waveform, the dual-wavelength shape is maintained with negligible variation by adjusting the λ/2 waveplate after collimator 2 when the dual-wavelength emission is in operation. Meanwhile, the dependences of the single-wavelength and dual-wavelength emission on pump power is also investigated. There are no signs of optical wave breaking or multiple pulses operation in our laser oscillator due to the limitation of the available pump power. And in most cases, the laser is operated in single-wavelength emission.

**Figure 5:**
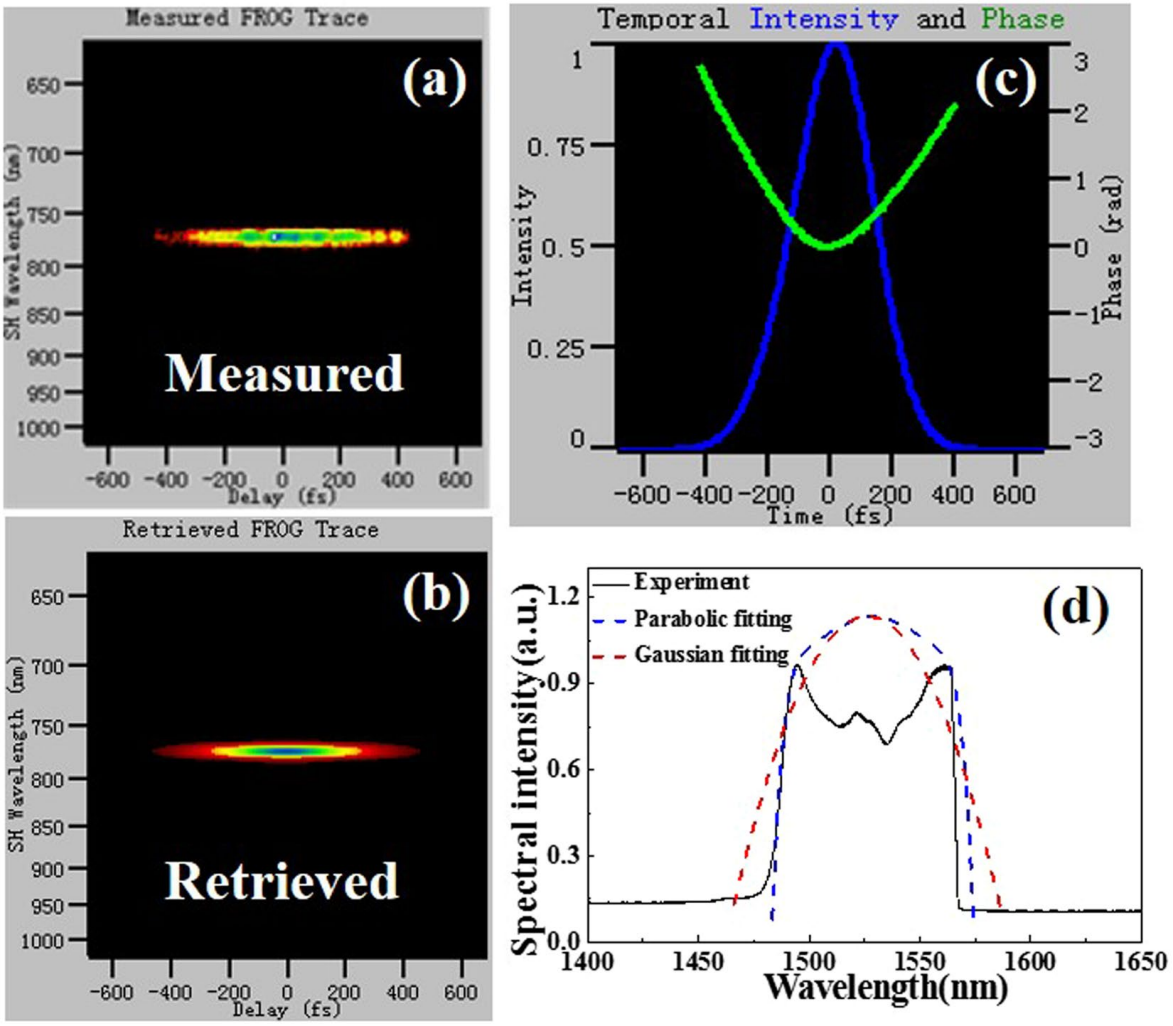
(**a**) Measured FROG trace, (**b**) retrieved FROG trace, (**c**) pulse and phase in time domain retrieved from FROG trace, and (**d**) spectrum of the short wavelength when the laser is operated in dual-wavelength emission.

**Figure 6:**
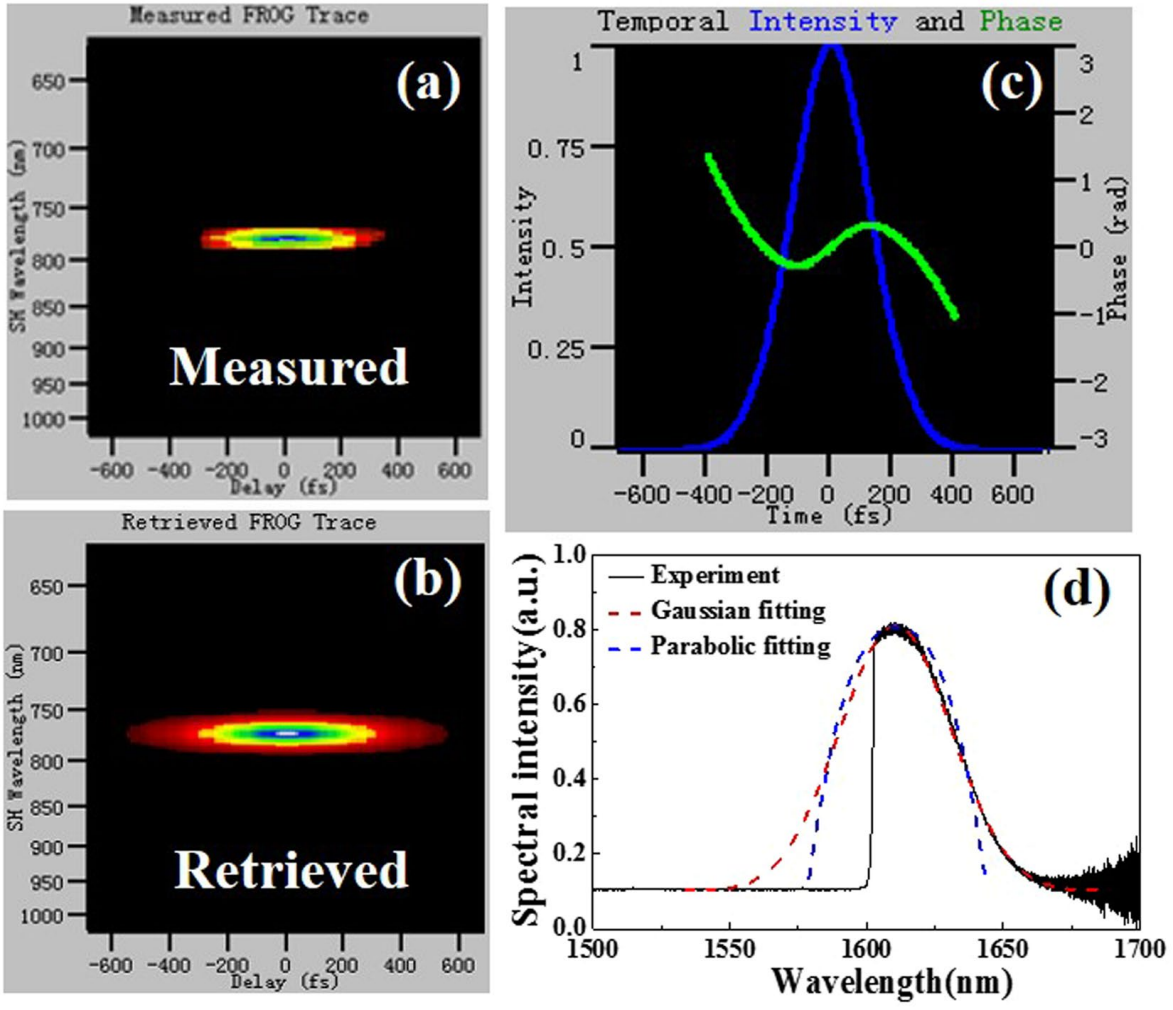
(**a**) Measured FROG trace, (**b**) retrieved FROG trace, (**c**) pulse and phase in time domain retrieved from FROG trace, and (**d**) spectrum of the long wavelength when the laser is operated in dual-wavelength emission.

The output dual-wavelength spectrum can be resolved into two independent spectra by exploiting two custom-made spectrum filters (a short-pass filter and a long-pass filter). The measured and retrieved FROG traces of the short wavelength spectrum are illustrated in Fig. 5(a) and (b), respectively. Figure 5(c) presents the pulse and phase in time domain with a pulse duration of 279.8 fs. The quadratic form in the phase indicates a linear chirp of the pulse, which is a typical characteristics of a self-similar parabolic pulse. The corresponding spectrum (Fig. 5(d)) shows a FWHM of about 78.5 nm (from 1488.3 nm to 1566.8 nm, the valley at 1530 nm is due to the ground state absorption of the long gain fiber). A parabolic function fits the spectrum well especially at the leading edge (blue dashed line in Fig. 5(d)). We thus claim that the pulses corresponding to the short wavelength spectrum are similaritons. Figure 6(a) and (b) are the measured and retrieved FROG traces of the long wavelength spectrum, respectively. Figure 6(c) presents the pulse and phase in time domain with a pulse duration of 313.7 fs. The absence of resonant sidebands and the significant chirp of the output pulses indicate that the laser operates in the stretched-pulse regime. The dual-wavelength pulses reported here are significantly shorter than the picosecond pulses previously observed in other dual-wavelength mode-locked fiber lasers^20–27^. Figure 6(d) provides the long wavelength spectrum with a FWHM of about 30.2 nm (from 1602.4 nm to 1632.6 nm). The Gaussian function (red dashed curve in Fig. 6(d)) fits the measured spectrum better than the parabolic function (blue dashed curve in Fig. 6(d)) at pulse trailing edges, indicating that the pulse is a dispersion-managed soliton rather than a self-similar soliton. Therefore, dual-wavelength mode-locking with different operating regimes are observed in a NPE-based broadband Er-doped fiber laser at near zero dispersion.

**Figure 7:**
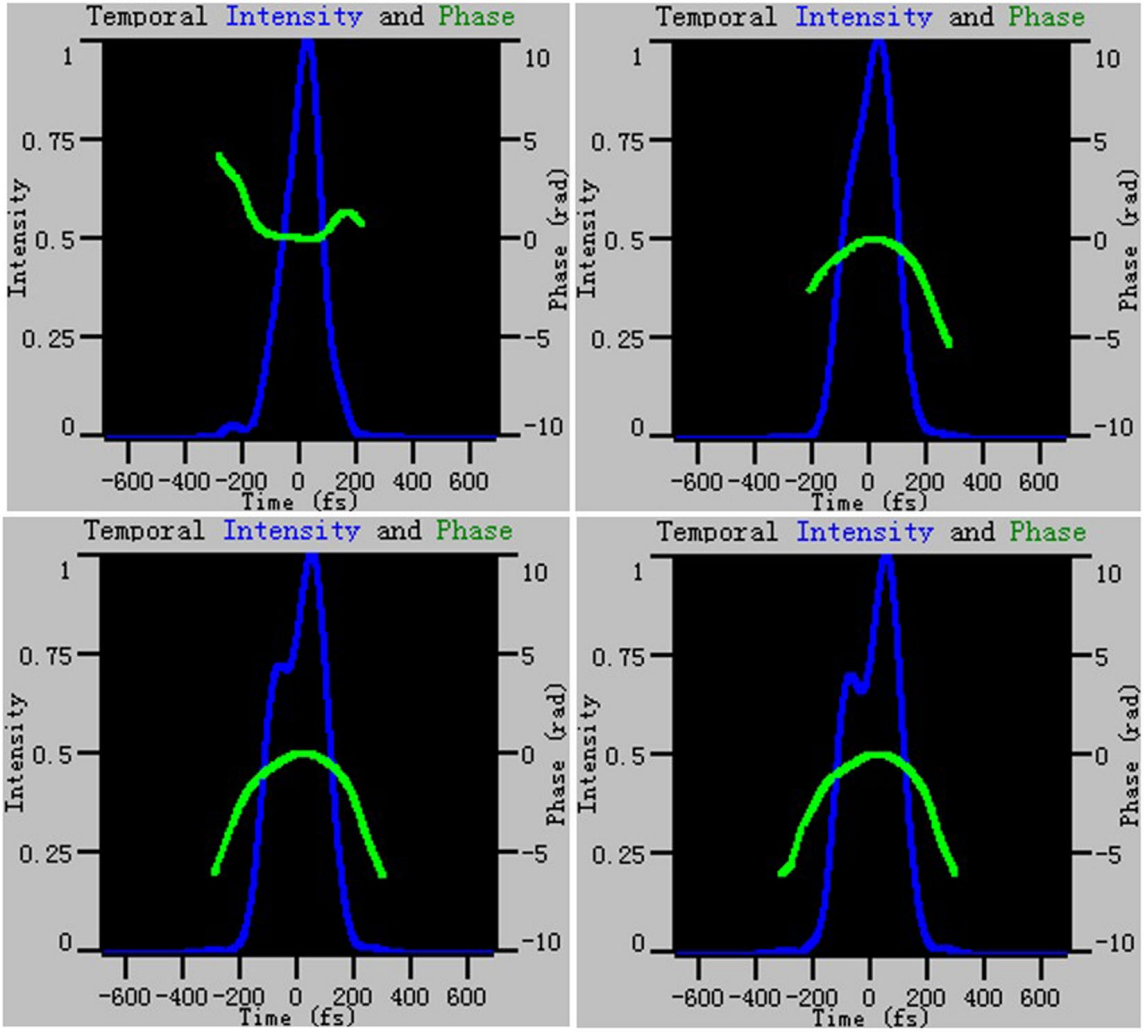
Evolution of the pulse waveform and phase characteristics of the fiber laser by adjusting the quarter waveplate before collimator1 in one direction.

The formation of the dual-wavelength emission results from an excessive accumulation of nonlinear phase and a resulting overdrive of the NPE saturable absorption. According to the NPE transmission model^17^, NPE introduces a varying polarization state across the pulse, resulting in a NPE switch carving out a section of the spectrum and allowing longer and shorter wavelengths to transmit. By adjusting the intracavity waveplates, the power stored in the laser cavity is changed with the variety of polarization dynamics. The high peak power combined with a chirped broadband pulse leads to the overdrive of the NPE switch. At the moment, the leading edge and trailing edge of the spectrum can experience either the same or different cavity loss due to the NPE switch effect. In a particular case, both the leading and trailing edges experience quite low loss, while the pulse peak undergoes high loss. As a result, the pulse peak is rejected from the NPE switch, whereas the leading and trailing edges experience maximum transmission. Then, within the effective gain bandwidth, the two spectral components are sufficiently separated and the dual-wavelength operation is obtained. In our experiments, we find that the dual-wavelength emission is very sensitive to λ/4 waveplates setting and pump power, indicating that the nonlinear phase plays a key role. Figure 7 depicts the evolution of the pulse waveform and phase characteristics of the fiber laser during the optimization of the dual-wavelength emission by adjusting the λ/4 waveplate before collimator 1 in one direction. The laser is first operated at single wavelength emission, with appropriate adjustment of λ/4 waveplate angle before collimator 1, the laser can operate at dual-wavelength emission with distinct central wavelengths, and each mode-locked spectrum corresponds to an independent pulse. In our fiber laser, dual-wavelength pulses are not completely separated due to the limitation of the available pump power. This view has also been confirmed in ref. 27, which shows that the multi-wavelength behavior can be induced by variations in the ellipticity of the circular polarization state and an overdrive of the NPE by numerical simulation based on the generalized vector nonlinear Schrödinger equation. Meanwhile, the single circulating pulse mechanism eliminates the effect of group velocity mismatch between two independent pulses, and hence maintains a fixed temporal separation between the pulses corresponding to different wavelengths at the laser output.

It has been indicated that the output characteristics of the pulse laser can be strongly influenced by the cavity dispersion when the net cavity dispersion approaches zero^28^. In our NPE-based mode-locked laser, the optical spectrum of the dual-wavelength emission is broad with a FWHM of 145.4 nm (from 1488.1 to 1633.5 nm). But the net cavity GVD is approximately maintained at +0.0048 ps^2^ only at 1550 nm wavelength. It is reasonable to argue that the operating regime of the dual-wavelength emission fiber laser is still influenced by different dispersion mechanisms within the whole wavelength range. According to the fiber parameters in Table 1, the net cavity GVD are +0.0056 ps^2^ at 1533 nm for the short wavelength spectrum which locates in the dispersion range of self-similar regime and +0.0023 ps^2^ at 1615 nm for the long wavelength spectrum which locate in the dispersion range of stretched-pulse regime, respectively. Therefore, different net cavity GVD, the strongly chirped pulse, the broadband spectra, and the NPE, concurrently lead to the two pulses of dual-wavelength emission with different formation mechanisms. The short wavelength laser at 1533.1 nm operates in the self-similar regime and exhibits a parabolic pulse waveform; while the long wavelength laser at 1615.4 nm operates in the stretched-pulse regime and shows a Gaussian-like pulse waveform. Dual-wavelength emission with different pulse waveforms in our fiber laser indicates that two types of pulses with different laser operating regimes can not only coexist in one ring cavity but also be generated simultaneously. This demonstration not only reveals comprehensive pulse formation dynamics (e.g., coexistence between different mode-locking regimes), but also can be potentially used for ultrafast lasers with wide optical spectra and short pulses.

## Conclusions

In conclusion, we have experimentally demonstrated that dispersion-managed solitons and similaritons can be launched simultaneously in a NPE-based Er-doped fiber ring laser by fine-tuning the waveplates to change the polarization state in the cavity. The net GVD of the laser oscillator is managed to be near zero, which locates in the transition region between stretched-pulse and self-similar regimes. When the laser is operated in dual-wavelength emission, the output pulses corresponding to the two wavelengths exhibit Gaussian-like and parabolic-like waveforms, respectively, indicating that the laser can simultaneously operate in stretched-pulse and self-similar regimes. The physics behind the dual-wavelength operation can be attributed to an overdriven NPE switch acting on a chirped broadband pulse and different dispersion mechanisms. These findings not only reveal how multi-wavelength operation with different pulse waveforms can be achieved, but also provide a good foundation for comprehensively studying pulse formation dynamics in laser cavities.
